# Association between self-reported walking speed and calcaneal stiffness index in postmenopausal Japanese women

**DOI:** 10.1186/s12877-020-01858-4

**Published:** 2020-11-11

**Authors:** Yoshihito Tomita, Kazuhiko Arima, Satoshi Mizukami, Ritsu Tsujimoto, Shin-ya Kawashiri, Takayuki Nishimura, Takuhiro Okabe, Natsumi Tanaka, Yuzo Honda, Kazumi Nakahara, Naoko Yamamoto, Izumi Ohmachi, Hisashi Goto, Maiko Hasegawa, Youko Sou, Itsuko Horiguchi, Mitsuo Kanagae, Yasuyo Abe, Fumiaki Nonaka, Mami Tamai, Hirotomo Yamanashi, Yasuhiro Nagata, Atsushi Kawakami, Takahiro Maeda, Kiyoshi Aoyagi

**Affiliations:** 1 School of Rehabilitation, Department of Physical Therapy, Tokyo Professional University of Health Sciences, Tokyo, Japan; 2 Department of Public Health, Nagasaki University Graduate School of Biomedical Sciences, 1-12-4 Sakamoto, Nagasaki, 852-8523 Japan; 3 Department of Rehabilitation, Nishi-Isahaya Hospital, Isahaya, Japan; 4 Department of Orthopedic Surgery, Nagasaki University Graduate School of Biomedical Sciences, Nagasaki, Japan; 5 Department of Community Medicine, Nagasaki University Graduate School of Biomedical Sciences, Nagasaki, Japan; 6 Department of Human Science, Faculty of Design, Kyushu University, Fukuoka, Japan; 7 Department of Rehabilitation, Faculty of Health Sciences, Tokyo Kasei University, Saitama, Japan; 8 Faculty of Health Science, Kumamoto Health Science University, Kumamoto, Japan; 9 Department of Health Science, Faculty of Medicine Kagoshima University, Kagoshima, Japan; 10 Department of Health Science, Nagasaki University Graduate School of Biomedical Sciences, Nagasaki, Japan; 11 Ken-Hoku Health Care Office, Nagasaki, Japan; 12 Medical Policy Division, Nagasaki Prefectural Government, Nagasaki, Japan; 13 Ken-Nan Health Care Office, Nagasaki, Japan; 14 Center for Public Relations Strategy, Nagasaki University, Nagasaki, Japan; 15 Department of island and rural medical research, Nagasaki University Graduate School of Biomedical Sciences, Nagasaki, Japan; 16 Department of Immunology and Rheumatology, Nagasaki University Graduate School of Biomedical Sciences, Nagasaki, Japan; 17 Department of Clinical Medicine, Institute of Tropical Medicine, Nagasaki University, Nagasaki, Japan; 18 Center for Comprehensive Community Care Education, Nagasaki University Graduate School of Biomedical Sciences, Nagasaki, Japan

**Keywords:** Self-reported walking speed, Calcaneal stiffness index, Postmenopausal women

## Abstract

**Background:**

Osteoporosis and related fractures, a worldwide public health issue of growing concern, is characterized by compromised bone strength and an increased risk of fracture. Here we show an association between self-reported walking speed and bone mass among community-dwelling postmenopausal Japanese women aged 50 years and older.

**Design; cross-sectional study:**

Setting and Participants; The survey population included 1008 postmenopausal women 50–92 years of age residing in rural communities.

**Methods:**

Self-reported walking speed was ascertained by asking the participants: “Is your walking speed faster than others of the same age and sex?” to which participants responded “yes (faster)” or “no (moderate/slower).” Calcaneal stiffness index was measured.

**Results:**

Women with a faster self-reported walking speed were younger and had a lower BMI, higher stiffness index, and higher grip strength than women with a slower walking speed. Multiple linear regression analysis adjusted for age, BMI, grip strength, comorbidity, current smoking, and alcohol drinking status showed a significant association between faster self-reported walking speed and higher calcaneal stiffness index (*p* <  0.001).

**Conclusions:**

Our findings suggest that questionnaires of walking speed may be useful for predicting bone mass and that a fast self-reported walking may benefit bone health in postmenopausal women.

## Brief summary

Faster walking speed was related to higher calcaneal stiffness index after adjusting for age, BMI, grip strength, comorbidities, smoking, and alcohol drinking in postmenopausal Japanese women.

## Background

Osteoporosis and related fractures comprise a major life-threatening issue for elderly individuals worldwide [1]. This issue is particularly serious in Japan, which has the highest life expectancy worldwide. The prevalence of osteoporosis is significantly higher in women than in men [2]. Therefore, maintaining or improving the bone quality of elderly women has become of increased interest in recent years.

A low bone mass is associated with osteoporosis, defined as a skeletal disorder characterized by compromised bone strength predisposing a person to an increased risk of fracture. It is important to acknowledge a common misconception that osteoporosis is always the result of bone loss [3]. Quantitative ultrasonography (QUS) is reportedly useful for evaluating bone mass. Although the diagnosis of osteoporosis is commonly based on measurements taken on dual energy X-ray absorptiometry, which has been considered the gold standard, [4, 5] QUS is now widely used because it is a non-ionizing and low-cost method against osteoporosis [6]. Moreover, also phalangeal quantitative ultrasound have been recently associated to fracture risk and poor bone health [7, 8].

Gait speed may be a simple and accessible indicator of health in the older person [9]. Moreover, it is featured in emerging consensus definitions of sarcopenia and frailty [10, 11]. A slower usual walking speed is a risk factor for adverse outcomes, including decreased activities of daily living, [12] falls and institutionalization, [13] and fracture and cognitive decline [14].

However, measuring walking speed requires observer training and more time than simply asking a person to self-report their customary walking speed. Moreover, not all research studies involve face-to-face contact with study participants and not all research and clinical settings have the space to set up a walking course. In addition, an older person may temporarily lack the ability to complete a walking assessment during periods of acute illness, injury, or hospitalization. Therefore, an alternative approach to characterizing customary walking speed would be valuable in settings in which direct measurements are not feasible.

Increased stress on the bone during physical activity could stimulate maintained or increased bone density [15]. However, the role of customary physical activity such as walking has remained inconclusive [16]. Walking speed was shown to contribute to bone health in women over 60 years of age [17]. Increased walking speed increases the vertical ground reaction force [18]; thus, daily brisk walking might contribute to bone health [17]. Self-reported walking speed is strongly associated with measured walking speed among community-dwelling people [19]. We hypothesized that fast self-reported walking speed could increase the vertical ground reaction force and increase bone stress, which may positively affect bone health.

To date, the effect of self-reported walking speed on bone mass has not been well studied. Thus, this study aimed to assess the association of self-reported walking speed with bone mass among community-dwelling Japanese postmenopausal women aged 50 years and older.

## Methods

Participants voluntarily enrolled in the study. Written consent forms were available in Japanese to ensure comprehensive understanding, and each participant provided informed consent. This study was approved by the Ethics Committee for Human Use of Nagasaki University (project registration no. 14051404).

The survey population included 1012 postmenopausal women 50–92 years of age residing in rural communities in Goto city in western Japan who underwent a general medical check-up in 2014 and 2016 as recommended by the Japanese government. All participants had sufficient cognitive function to complete the questionnaire.

Trained interviewers obtained information about the participants’ clinical characteristics. Comorbidity data including heart disease, lung disease, stroke, or diabetes mellitus (DM) were also collected.

Self-reported walking speed was ascertained by asking the participants: “Is your walking speed faster than others of the same age and sex?” Participants responded “yes (faster)” or “no (slower).” This was one of the four walking speed questions related to true walking speed [20].

Broadband ultrasound attenuation (BUA; dB/MHz) and speed of sound (SOS; m/s) through the right calcaneal bone were measured using an Achilles ultrasound bone densitometer (GE Lunar Corp., Madison, WI, USA). Calcaneal stiffness index, a function of BUA and SOS, was automatically calculated by the scanner software according to the following formula: [21] Stiffness index = (0.367 × BUA) + (0.28 × SOS) - 420.

Patient body weight and height while wearing light clothing were measured using an automatic body composition analyzer (BF-220; Tanita, Tokyo, Japan), and body mass index (BMI) (kg/m^2^) was calculated.

Grip strength was recorded as the grip strength from two measurements performed with each hand using a handgrip dynamometer (Smedley, Matsumiya Ika Seiki Seisakujo, Tokyo, Japan); the maximum value as used in the analysis.

### Statistical analysis

Women with missing values for any variables were excluded from analysis, leaving 1008 women for our analysis. Comparisons of age, BMI, stiffness index, grip strength, comorbidities, current smoking, and current alcohol drinking between subjects with faster versus slower self-reported walking speeds were performed using Student’s t-test for continuous variables or the chi-square test for nominal variables.

Multiple linear regression analysis was used to explore the effects of faster self-reported walking speed on calcaneal stiffness index with the adjustment for age, BMI, grip strength, comorbidity, current smoking, and current alcohol drinking.

Values of *p* <  0.05 were considered significant. All statistical analyses were performed using SPSS software version 20 (SPSS Inc., Chicago, IL, USA).

## Results

The subjects’ characteristics are shown in Table 1. The mean age, BMI, stiffness index, and grip strength were 69.1 years, 22.8 kg/m^2^, 66.3, and 20.8 kg, respectively. Sixteen percent of the cohort had at least one comorbidity, 2% were current smokers, and 12% were current drinkers. The prevalence of faster self-reported walking speed was 41.5% (418/1008).

**Table 1:** Participants’ characteristics (*N* = 1008)

Variable	Mean ± standard deviation	Range
Age (years)	69.1 ± 8.4	50–92
Body mass index (kg/m^2^)	22.8 ± 3.4	14.3–40.7
Stiffness index	66.3 ± 12.9	29.2–119.6
Grip strength (kg)	20.8 ± 5.2	2.5–35.3
	Number (%)	
One or more comorbidity^a^	160 (15.9)	
Current smoking	22 (2.2)	
Current alcohol drinking	119 (11.8)	
Faster self-reported walking speed	418 (41.5)	

^a^Heart disease, lung disease, stroke, or diabetes mellitus

Table 2 compares the variables between subjects with faster versus slower self-reported walking speeds. Women with a faster self-reported walking speed were younger and had a lower mean BMI, higher stiffness index, and higher grip strength than those with a slower self-reported walking speed (*p* <  0.05).

**Table 2:** Comparison of variables between subjects with faster versus slower self-reported walking speed (*N* = 1008)

Variable	Faster (*n* = 418)	Slower (*n* = 590)	*P* value^b^
Age (years)	68.4 ± 8.0	69.6 ± 8.6	0.030
Body mass index (kg/m^2^)	22.3 ± 3.1	23.1 ± 3.6	< 0.001
Stiffness index	68.5 ± 12.4	64.8 ± 13.1	< 0.001
Grip strength (kg)	21.6 ± 4.8	20.2 ± 5.3	< 0.001
One or more comorbidity^a^	50 (12.0)	110 (18.6)	0.004
Current smoking	8 (1.9)	14 (2.4)	0.623
Current alcohol drinking	58 (13.9)	61 (10.3)	0.086

Data are shown as mean ± standard deviation or number (%)

^a^Heart disease, lung disease, stroke, or diabetes mellitus

^b^Student’s t-test was used to analyze continuous variables; the chi-square test was used to analyze categorical variables

Multiple linear regression analysis adjusted for age, BMI, grip strength, comorbidities, current smoking, and current alcohol drinking showed a significant association between faster self-reported walking speed and higher calcaneal stiffness index (*p* <  0.001) (Table 3).

**Table 3:** Association between faster self-reported walking speed and calcaneal stiffness index: results of multiple regression analysis adjusted for age, body mass index, grip strength, comorbidities, current smoking, and current alcohol drinking (*N* = 1008)

Variables	B	95% confidence interval	*P* value
Faster self-reported walking speed	2.936	1.471–4.401	< 0.001
Adjusted factors			
Age (years)	−0.564	- 0.665 – − 0.462	< 0.001
Body mass index (kg/m^2^)	0.434	0.223–0.645	< 0.001
Grip strength (kg)	0.283	0.120–0.447	0.001
One or more comorbidity^a^	1.057	−0.939 – 3.052	0.299
Current smoking	- 0.244	- 5.150 – 4.662	0.922
Current alcohol drinking	- 0.986	- 3.233 – 1.260	0.389

^a^Heart disease, lung disease, stroke or diabetes mellitus

## Discussion

### Stiffness index and self-reported walking speed

Women with a faster self-reported walking speed had a higher mean stiffness index than those with a slower self-reported walking speed after the adjustment for age, BMI, grip strength, comorbidities, current smoking, and current alcohol drinking. A previous study showed that self-reported walking speed is a good marker of measured walking speed [19]. Self-reported walking speed is a useful measurement when the use of actual walking speed is not feasible. Cross-sectional studies have reported that calcaneus bone quality in elderly women is significantly associated with walking speed, stride length, and total number of steps [17, 22]. Increased walking speed caused a significant increase in activities of the lumbar erector spine, biceps femurs, and medial gastrocnemius; lumbar motion; and vertical ground reaction force in the loading response and mid-stance phases [18]. Therefore, women with a faster self-reported walking speed may be placing an increased mechanical strain on bone due to an increased vertical ground reaction force and consequently gain bone mass.

### Stiffness index and age

In this study, older age was associated with a lower stiffness index in postmenopausal women. This result supports that of a previous study that calcaneus stiffness index significantly decreased with aging [23]. Gregg et al. reported that, in addition to age-related bone loss, menopause-related acceleration of a loss of stiffness index appears to occur in the perimenopausal period [24]. This result suggests that measuring and maintaining bone mass is important in postmenopausal women.

### Stiffness index and BMI

The present study showed that a higher BMI was associated with a higher stiffness index in postmenopausal women. A higher BMI was associated with a higher stiffness index, similar to previous reports [23]. However, since obesity (a high BMI) is a major risk factor for cardiovascular disease and DM, maintaining a healthy BMI is recommended to support general health.

### Stiffness index and grip strength

In this study, a stronger grip strength was associated with a higher stiffness index in postmenopausal women. Previous studies demonstrated a relationship between stronger grip strength and a higher stiffness index [25]. Poor physical performance in elderly people may be reduced due to mechanical strain on bone resulting from reduced physical activity, [26] leading to a bone mass reduction. These results suggest a strong role of maintaining muscular strength for preventing bone loss in healthy and functionally independent elderly people.

### Bone mass and comorbidities (heart disease, lung disease, stroke, DM)

Patients with chronic heart failure had a lower reported bone mass than those without it [27]. The prevalence of osteoporosis is reportedly higher in patients with chronic obstructive pulmonary disease than in healthy subjects. Bone mass losses were greatest among patients with severe lung disease [28]. At 1 year of follow-up, a decreased bone strength index was reported in chronic stroke patients [29]. Several factors reportedly affect bone quality (and mass) in patients with DM, mainly due to reduced bone turnover [30]. Although our multiple linear regression analysis adjusted for such comorbidities, no significant difference was noted. Further studies, including other potential confounders, are needed to clarify the associations between comorbidities and bone mass.

### Stiffness index and smoking and alcohol drinking

Several studies indicated that smoking and alcohol consumption influence bone loss, although others did not confirm these findings [31]. In the present study, current smoking and current alcohol drinking was selected as explanatory variables on stiffness index in the multiple regression model. Few subjects were current smokers (2.1%) or current drinkers (11.9%). Thus, smoking and alcohol consumption appeared not to have a major influence on stiffness index in this study.

### Limitations

This study has several limitations. First, because actual walking speed was not measured, the relationship between actual walking speed and self-reported walking speed could not be examined directly in this subject. Second, because this study was conducted in a cross-sectional setting, these results do not show a causal relationship. Longitudinal studies are required to establish causal relationships between self-reported walking speed and bone mass. Third, the study subjects were community-dwelling residents who voluntarily attended a health examination, contributing to selection bias. It might suggest that the subject’s awareness of health may be higher than that in non-attendants. Therefore, our results can’t be adapted to the general population. Fourth, the present results were obtained from only Japanese women; therefore, it is not possible to extrapolate the results to men or individuals of other ethnicities. Fifth, potential confounders that can affect self-reported walking speed and decreased bone mass, such as a sedentary lifestyle, vitamin D deficiency, low calcium intake, secondary hyperparathyroidism, peripheral nerve dysfunction, disorders of skeleton, vertebral osteoarthrosis, kyphosis, current medications, years after menopause, and renal insufficiency, were unavailable for participants in this study.

## Conclusions

Our findings suggest that questionnaires of walking speed may be useful for predicting bone mass and that a fast self-reported walking speed may benefit bone health and contribute to a high bone mass in postmenopausal women.

## Data Availability

The datasets obtained and/or analysed during the current study are not publicly available as the datasets are highly detailed and we are planning to publish more papers using the same dataset. The datasets are available from the corresponding author on reasonable request.

## Abbreviations

BUA: Broadband ultrasound attenuation
BMI: Body mass index
DM: Diabetes mellitus
QUS: Quantitative ultrasonography
SOS: Speed of sound
